# Adsorption of Hydrogen Sulfide on Activated Carbon Materials Derived from the Solid Fibrous Digestate

**DOI:** 10.3390/ma16145119

**Published:** 2023-07-20

**Authors:** Evangelia Choleva, Anastasios Mitsopoulos, Georgia Dimitropoulou, George Em. Romanos, Evangelos Kouvelos, George Pilatos, Konstantinos Beltsios, Stylianos Stefanidis, Angelos Lappas, Themistoklis Sfetsas

**Affiliations:** 1 QLAB Private Company, Research & Development, Quality Control and Testing Services, 57008 Thessaloniki, Greece; choleva.evangelia@gmail.com (E.C.); tmitsop@ergoplanning.gr (A.M.); g.dimitropoulou@q-lab.gr (G.D.); 2 Institute of Nanoscience and Nanotechnology, National Center of Scientific Research “Demokritos”, 15310 Athens, Greece; g.romanos@inn.demokritos.gr (G.E.R.); v.kouvelos@inn.demokritos.gr (E.K.); g.pilatos@inn.demokritos.gr (G.P.); 3 Department of Materials Science and Engineering, University of Ioannina, 45110 Ioannina, Greece; kgbelt@mail.ntua.gr; 4 Biogas Lagada SA, 677 Parcel, Kolchiko, 57200 Lagadas, Greece; 5 School of Chemical Engineering, National Technical University of Athens, Zografou Campus, 9, Iroon Polytechniou Str., Zografou, 15780 Athens, Greece; 6 Laboratory of Environmental Fuels/Biofuels and Hydrocarbons, Chemical Process and Energy Resources Institute, CERTH, 57001 Thessaloniki, Greece; s.stefanidis@certh.gr (S.S.); angel@cperi.certh.gr (A.L.)

**Keywords:** activated carbon, hydrogen sulfide, biogas, physical adsorption, micropores, biogas, solid fibrous digestate, carbon dioxide, mesopores

## Abstract

The goal of this work is to develop a sustainable value chain of carbonaceous adsorbents that can be produced from the solid fibrous digestate (SFD) of biogas plants and further applied in integrated desulfurization-upgrading (CO_2_/CH_4_ separation) processes of biogas to yield high-purity biomethane. For this purpose, physical and chemical activation of the SFD-derived BC was optimized to afford micro-mesoporous activated carbons (ACs) of high BET surface area (590–2300 m^2^g^−1^) and enhanced pore volume (0.57–1.0 cm^3^g^−1^). Gas breakthrough experiments from fixed bed columns of the obtained ACs, using real biogas mixture as feedstock, unveiled that the physical and chemical activation led to different types of ACs, which were sufficient for biogas upgrade and biogas desulfurization, respectively. Performing breakthrough experiments at three temperatures close to ambient, it was possible to define the optimum conditions for enhanced H_2_S/CO_2_ separation. It was also concluded that the H_2_S adsorption capacity was significantly affected by the restriction to gas diffusion. Hence, the best performance was obtained at 50 °C, and the maximum observed in the H_2_S adsorption capacity vs. the temperature was attributed to the counterbalance between adsorption and diffusion processes.

## 1. Introduction

Biogas use as a source of renewable energy has increased over the years and the demand for economically attractive methods for biogas upgrading is of growing interest. Hydrogen sulfide is one of the main contaminants that needs to be removed before the biogas stream enters the CHP due to its corrosive effect. One of the most effective, low cost and easy-to-maintain methods for H_2_S removal is the in situ biological reduction, implemented either by adding iron salts/oxides or by air dosing to the digester’s slurry where biological anaerobic oxidation of H_2_S to elemental sulfur and sulfates happens by *Thiobacilus* bacteria. Adding iron salts/oxides is a very effective practice in reducing high H_2_S levels down to 100–200 ppmv, but it fails to maintain a stable level of H_2_S. Most regularly practiced is the use of liquid FeCl_2_, while Fe(OH)_3_, Fe(OH)_2_ and ferrous chloride FeCl_3_ can also be involved in their solid form. The method of dosing 3–6% air to the biogas ratio can achieve 80–99% H_2_S reduction, down to 20–100 ppm H_2_S [1], while the oxygen content will be 0.5–1.8% per volume. The second most frequently applied process is adsorption, which entails trapping pollutants on a solid with a high surface area (activated carbon or crystalline material with high porosity, e.g., zeolites, silica gel, activated alumina), holding the pollutants through physical (weak) attraction forces or via chemical bonding. Adsorption is one of the most competitive technologies for precision desulfurization because it is simple and effective (>99%). The most competitive adsorbents for H_2_S biogas removal are impregnated activated carbons and iron oxides [1,2]. Adsorption systems are typically suitable for flow rates between 10 and 10,000 m^3^/h and pollutant concentrations between 0.1 and 8 g/m^3^ [3,4]. Impregnated activated carbons are preferably applied when it is necessary to significantly reduce or eliminate the concentration of H_2_S. This is because, in addition to the physical adsorption, activated carbons provide catalytic sites for oxidation to elemental sulfur and sulfate, thus enhancing the removal capacity of H_2_S. The activated carbons (ACs) must maintain a content of 20–30% moisture and the required volume of oxygen [5]. When the levels of H_2_S in the feed stream are high (>3000 ppmv), the adsorptive and catalytic sites are saturated, making periodic regeneration of the AC adsorbents necessary. Impregnated products usually exhibit enhanced H_2_S removal capacity, from a normal 10–20 kg H_2_S/(m^3^ C) for virgin carbon to 120–140 kg H_2_S/(m^3^ AC) for activated carbon. The cons are that the regeneration of those used in the process ACs is not sustainable and, consequently, the spent carbon must either be landfilled, applying to policy on nitrogen pollution (EU Nitrates Directive (1991) [6]), or re-impregnated, adding up to the logistic costs [7].

There is thereby a new interest field that emerged in using the biomass, even better the solid fibrous digestate (SFD), to produce the ACs on-site for the desulfurization of the biogas. In this way, a waste product (SFD) is transformed into an effective adsorbent that can be used either on-site, creating a closed loop value chain within the biogas plant, or implemented in other gas separation and wastewater treatment processes, achieving the effective integration between value chains [8,9].

To date, activated carbon adsorbents for biogas desulfurization have been prepared and studied using the common raw materials usually involved to yield ACs. Guo et al. [10] prepared chemically and physically activated carbons based on palm and coconut shells and investigated the different mechanisms of H_2_S adsorption, physisorption, chemisorption and H_2_S oxidation, depending on the activation agent, using H_2_O, CO_2_, ΚOH and H_2_SO_4_ and concluded that chemical activation has better dynamic adsorption performances. Longer breakthrough times and prolonged exhaustion times seem to increase the H_2_S adsorption capacities. Javier et al. [11] investigated the production of AC from barley straw via a physical activation method with CO_2_ and steam and concluded that the optimal conditions for the activation stage with CO_2_ were at 800 °C and a residence time of 1 h and at 700 °C and a hold time of 1 h when H_2_O is the activating agent [12,13,14]. The maximum BET surface area and micropore volume achieved by carbon dioxide activation were 789 m^2^/g and 0.3268 cm^3^/g, while for steam activation, they were 552 m^2^/g and 0.2304 cm^3^/g, representing an increase on both values of more than 42% for the case of activation with carbon dioxide. Those ACs functionalized with CO_2_ presented a well-developed micropore structure compared to the lower degree of microporosity endowed in the steam-activated carbons. Calderon et al. achieved 94% retention of H_2_S using AC as an adsorbent. This AC was derived from the sludge of a wastewater treatment plant while activation was performed with 1 M KOH for 20 min at 700 °C. Furthermore, simulation studies predicted that the retention of H_2_S could be enhanced to 96% [15], for an activation temperature of 635 °C and KOH molarity of 2.6 M. Cattle manure-based activated carbon materials prepared with a flow rate of 1000 mL/min CO_2_ has a total feasible manufacturing cost of €0.97 per kg [16]. In this study, AC activated with increased CO_2_ flow reached a maximum of 408.36 m^2^/g in surface area and 0.0528 cm^3^/g in micro-pore volume and possessed a superior H_2_S adsorption capacity of 868.45 mg/g. Akhtar Hussain [17] investigated 2, 3, 6 and 9-ring carbon structured adsorbent surfaces using parameters such as the planar and non-planar mode and the surface defects. The 6-ring with the vacancy centrally located in non-planar mode illustrated the highest steric heat of sorption (q_st_) for H_2_S, while adsorption was performed with significant strength both on a non-defected and defected 3-ring model in planar mode. As a general outcome, the increase in the size and structure decreases the q_st_ and the most suitable configuration for the phenomenon to happen is central in a non-planar mode. Tangsathitkulchai et al. [18] investigated the equilibrium and kinetics of CO_2_ adsorption at 273 K by coconut shell activated carbon impregnated with sodium hydroxide (NaOH) and concluded that the maximum effective pore diffusivity occurred at an optimum NaOH loading of 180 mg/g, in line with the equilibrium adsorption results and achieved retention of CO_2_ up to 200 mg/g. Equilibrium isotherms of CO_2_ adsorption displayed an initial part of the Type I isotherm, with most adsorption taking place in micropores in the range of 0.7–0.9 nm by pore-filling mechanisms.

Many studies have shown that impregnated ACs are suitable adsorbents for many gases. Fomkin et al. [19] investigated the adsorption of hydrogen on KOH-activated ACs. Their study was conducted at the temperature range of 30 to 90 °C and pressures up to 200 bar, and the optimum conditions for enhanced adsorptivity of 11 mmol[H_2_]/g were found to be at 30 °C and 160 bar. Higher pressure favored the adsorption phenomenon, regardless. Nahum et al. [20] applied physical activation under CO_2_ atmosphere, at 850 °C for 2 h on carbon materials derived from cigarette butts and achieved a maximum adsorption capacity of 269 mg[C_6_H_6_O]/g of phenol at 10 °C, concluding that for phenol adsorption, the microporosity of the material is a crucial parameter. Moreover, ACs show good adsorbance behavior on the aqueous phase to remove toxic organic chemical compounds, such as benzene derivatives [21], and work well as heavy metal adsorbents [22]. Concerning the use of Solid Fibrous Digestate as a precursor material to produce activated carbons appropriate for the adsorption of on-site contaminants of the biogas production process, research oriented to phosphate adsorption on the liquid fraction of the digestate [23] concluded that the amount of solid digestate produced is sufficient for the removal of approximately 20% of the phosphate present. Biochar produced at 1000 °C pyrolysis temperature up to 100% recovery of phosphate solution at 50 mg/L or lower for higher phosphate concentration.

Further to the investigations on the development of highly efficient and selective for H_2_S, activated carbon adsorbents, there are also many studies focusing on the engineering part of the desulfurization process, elaborating either standalone processes or cascades that combine different processes such as adsorption on activated carbons and steel wool and absorption into aqueous solutions of amine, sodium hydroxide and calcium hydroxide [24,25]. The target of these studies is to instigate the overall process with the desired functionality, which can be either to enhance the CH_4_ content of biogas or to effectively remove H_2_S and CO_2_. Relevant reported results are presented in Table 1, concluding that the combination of calcium hydroxide (1 Molar) and steel wool (Fe and Zn elements) favors CO_2_ and H_2_S removal (max −44% and −97% respectively) while a combination of sodium hydroxide (1.5 Molar), activated carbon and steel wool favors CH_4_% content enhancement (up to +30% max) and CO_2_ removal (up to −41% max).

For other parameters for optimization related to the process conditions, it was shown that the biogas inlet pressure had a varying effect on the performance depending on the composition of the solvent (absorbent) and the type of adsorbent, whereas the amplification of the biogas feed flow rate had a negative effect on the targets of high CH_4_ content and effective CO_2_ removal [26,27,28]. On the contrary, H_2_S was favored for flow rates up to 10 LPM. While cascades of absorption and adsorption processes offer the flexibility to select among a great variety of solvents and adsorbents and to fine-tune the conditions toward achieving the required performance, they also present major difficulties related to the need to design and integrate completely different absorber and adsorber columns and the great variety of processes required for the regeneration of solvents and adsorbents along with the different frequencies of regeneration. These difficulties are showcased in Table 2.

Conclusively, the design of cascade processes integrating different solid adsorbents with tailor-made gas adsorption and separation capacity seems to be a more feasible solution for applications in biogas desulfurization and upgrading.

In this context, the present study achieves the dual target of developing effective adsorbents from the waste effluent of a biogas plant and further endowing them with enhanced CO_2_ separating or H_2_S separating capacity. Hence, the developed, in this work, activated carbons can be applied in stand-alone or cascade processes with the targets of desulfurizing biogas and enhancing its CH_4_ content. Starting from the solid fraction of digestate, pre-treatment and pyrolysis techniques are first optimized to achieve high yields of biochar (BC) along with a high external surface [29,30,31,32]. Furthermore, biochar is converted to activated carbon by applying a variety of physical and chemical activation methods with CO_2_, H_2_O and KOH, optimizing the functionalization conditions to further enlarge the carbon’s surface and the porosity and aiming for higher adsorption yields. Breakthrough experiments in fixed bed columns at different temperatures using real biogas mixtures are performed, and the obtained gas uptake and separation performances of the various ACs are scrutinized against their pore structural and surface chemistry properties. Conclusively, the outcome of this work is an optimized workflow that starts with SFD and ends up with a tailor-made adsorbent for either enhanced CO_2_ or H_2_S separation.

## 2. Experimental Procedures

### 2.1. Materials and Methods

The precursor material, namely solid fibrous digestate (SFD), used for the AC preparation was collected from a biogas plant in Greece (Biogas Lagada, Kolchiko, Greece), which uses mostly agricultural waste to feed the anaerobic digester (AD). The SFD is obtained from the whole digestate (WD) of the AD after separation in a screw filter press separator, followed by drying and sanitation. The total solids (TS) content of the SFD is between 90% and 95%.

**Figure 1:**
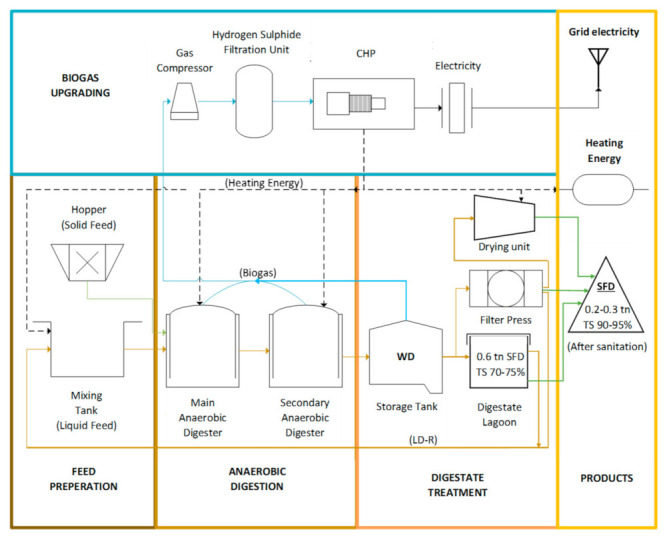
Biogas Plant Process Flowchart (SFD: solid fibrous digestate, WD: whole digestate, LD-R: liquid digestate recirculation, TS: total solids, CHP: combined heat and power).

The process flowchart of the biogas plant is presented in Figure 1, starting from the feed preparation stage where Solid Feed is mixed and then driven into the anaerobic digester along with the liquid feed from a mixing tank. The feedstock of the digesters is planned ahead, taking into account the biogas methane content and the digester’s biological equilibrium. The solid feedstock is mostly silage and other agricultural waste, and the liquid most common feedstock is cow and chicken manure, olive mill waste, meat processing waste, etc. Fresh feedstock enters the primary anaerobic digester (AD-1) while part of the previously digested material is led to the secondary one (AD-2) to remain there for a longer time, assisting the process of anaerobic digestion and maintaining the digester’s health. Part of the AD-2’s content is removed, leading to a storage tank and the material is then called Whole Digestate (WD). Sedimentation of Solid Digestate happens due to gravity and the supernatant Liquid Digestate (LD) is used as a recirculation liquid feed (LD-R) to the AD-1. The sediment of the Digestate Lagoon (Solid Digestate with TS 70–75%) is transferred to the screw filter press separator, followed by a drying unit, and after sanitation at 70 °C for 1 h, the Solid Fibrous Digestate (SFD) is attained, with TS 90–95%. Biogas produced in AD-1, AD-2 and the storage tank of the WD is led to a gas compressor, followed by a hydrogen sulfide filtration unit before entering the CHP engine. Electrical energy is turned into electricity and enters the national grid, while the heating energy produced on site is sufficient for the heating demands of the biogas plant (preheating the feed, heating the reactors, drying and sanitating the solid digestate).

In order to free the SFD precursor materials from their inorganic content, wash steps with 1% HNO_3_ solution were included (50 g SFD in 500 mL 1% HNO_3_ solution, at room temperature for 24 h). Thus, the inorganic matter was reduced from 12.9 wt.% to 4.6 wt.%, as shown in Table 3, whereas the carbon content increased from 42.2% for the SFD to 48.6% for the SFD washed with 1% HNO_3_ solution (15% increase). The resulting washed materials are further abbreviated as SFD-W. After washing, the SFD-W was dried at 105 °C for 24 h.

### 2.2. Equipment and Procedure for SFD Pyrolysis and BC Activation

#### 2.2.1. Pyrolysis of SFD and SFD-W

**Figure 2:**
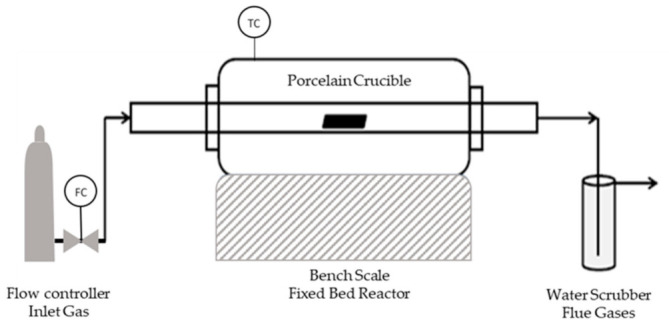
Pyrolysis Reactor.

The carbonization of the SFD and SFD-W was carried out via slow pyrolysis in a bench-scale fixed bed reactor (Figure 2), investigating the temperature parameter along with the residence time of the pyrolysis. A sample size of 5 g was carbonized at different temperatures (600–800 °C) and duration (30–120 min) (Table A1).

Achieving a higher pyrolysis yield was of high priority in this study since the overall yield of the whole process is relatively low. Thus, as long as the surface and pore characteristics are good, the pyrolysis parameters that favor the BC yield are preferred.

BC materials derived from the carbonization of the untreated SFD (BC-SFD) had a higher ash content (more than 33.60%), while the pretreated BC-SFD-W materials had less than half the ash content (up to 18.10%) (Table A2 and Table A3). This indicates that the removal of part of the inorganic compounds (~72%) from the precursor material with the HNO_3_ pretreatment is a highly important stage that must be common to any type of activated carbon development, as it allows the carbonization process to expand the carbon matrix.

#### 2.2.2. Activation of BC-SFD-W

BC activation was carried out via slow pyrolysis in a bench-scale fixed-bed reactor. Physical activation was carried out (a) with H_2_O (1 mL/min) at different temperatures (700–900 °C) and activation duration (15–90 min) (Table 4), with the water vapor flow controlled via a Bronkhorst CEM-System (Controlled Evaporation and Mixing); (b) with CO_2_ (50 mL/min) at 850 °C and activation duration of 150 min. For chemical activation with KOH, BC was mixed with solutions of KOH of different concentrations to achieve KOH/BC ratios of 1:4 (Table 5). After evaporation of the H_2_O, the mixture was activated at 600–800 °C for 30–120 min.

### 2.3. Test Rig for Biogas Breakthrough Experiments

**Figure 3:**
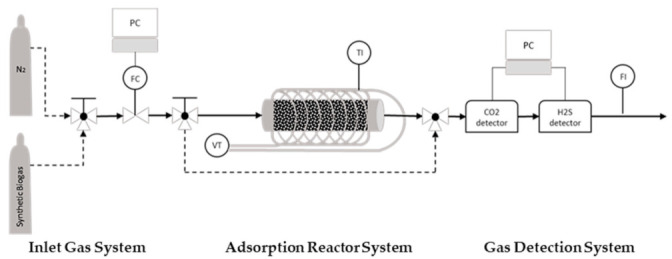
Adsorption Reactor; FC: Flow Controller; VT: Variac Transformer; TI: Temperature Indicator; FI: Flow Indicator.

The experimental system for obtaining the breakthrough curves consists of 3 parts; the inlet gas manifold, the fixed bed adsorber and a gas detection system (Figure 3). There are 2 bottles with gases, one with nitrogen and one with synthetic biogas (57% CH_4_, 42% CO_2_, 0.5% O_2_ and 500 ppm H_2_S). The inlet gas flow rates are controlled by a mass flow controller (MFC) and a three-way valve, which is installed upstream of the MFC to allow the selection of either a nitrogen or biogas mixture. The adsorber is a horizontal stainless steel tubular fixed bed, 7.9 cm long and 4.8 mm in the inner diameter. To initiate the experimental procedure, the activated carbon sample is positioned inside the adsorber tube, which is wall-heated by a heating mantle powered by a Variac Variable AC transformer. A temperature sensor is used to observe the temperature. Before each breakthrough experiment, the samples were regenerated under a nitrogen stream and a temperature of 250 °C. After lowering the temperature to the experimental value, the inlet stream is switched to by-pass, and the nitrogen gas is then switched to the biogas stream. As soon as the concentration of H_2_S is stabilized to the expected level (500 ppm), the inlet biogas stream is switched and allowed to pass through the adsorber containing the activated carbon sample. Thus, the adsorption phenomena happen and the breakthrough curves of H_2_S and CO_2_ can be logged via the software of the respective gas sensors. The concentrations of H_2_S and CO_2_ in the biogas stream escaping the adsorber column are quantitatively monitored using the SGX ECVW EK3 Electrochemical and Pellistor Gas Sensor Evaluation Kit and Rapidox Logger 7100 multigas analyzer, respectively. The experiments are conducted at an adsorption temperature of 25–70 °C, and the inlet gas flow is set to 50 mL/min. Measuring the real flow of the outlet biogas mixture downstream of the adsorber and taking into account the dimensional characteristics of the tubular reactor and the consistency of the synthetic biogas mixture, the pressure inside the reactor is calculated at an average of 0.044 millibars and the overflow rate at 220 cm/min (3.66 cm/s) on average. The adsorption temperature affects the kinematic viscosities of the gases in the mixture; thus, the flow characteristics vary, although slightly, for every single experiment (Table 6).

## 3. Results and Discussion

### 3.1. Biochar Yield

The BC yield of both untreated solid fibrous digestate and that pretreated with 1% HNO_3_, (Table A1) implies that pyrolysis at 600 °C achieves the highest yield compared to higher temperatures, while residence pyrolysis time seems to not affect the BC yield. It should be noted, however, that a shorter duration of pyrolysis has given the optimal yield of BC, e.g., 30 and 60 min for the BC produced from the non-washed SFD and the one washed with 1% HNO_3_ solution prior to the pyrolysis, respectively (Table 3). Since a decreased pyrolysis temperature and shorter carbonization time favor BC yield, the final preferred product is named BC-600 °C-30 min-SFD-W, which stands for BC produced from Pyrolysis at 600 °C for 30 min using the Solid Fibrous Digestate from the Biogas Lagada SA plant as a precursor material, pretreated with 1% HNO_3_ solution.

### 3.2. Elemental Analysis Results and Ash Content of BC

Elemental analysis shows that the carbon content is increased in the BC compared to its precursor material (Table A2 and Table A3). By increasing the pyrolysis temperature, the carbon content of the BC is increased while decreasing its hydrogen content. Likewise, though, this happens when extending the residence time of the pyrolysis. At low temperatures up to 200 °C the amount of hydrogen drops due to a decrease in the moisture content in the BC, which is also indicated by the reduction of the oxygen content. Up to 600 °C hydrogen content drops rapidly in the step of framework formation, presumably due to the completion of alkyl fragmentation. At higher temperatures up to 800 °C the nitrogen content drops and the same holds for hydrogen content. The ongoing lowering of the amount of nitrogen shows that further densification occurs, which mainly involves the elimination of nitrogen-containing side products. Furthermore, the processes occurring at 800 °C can be described as an ongoing condensation of the aromatic systems upon further elimination of elemental hydrogen and nitrogen.

### 3.3. Pore Structural Characteristics of the Developed BCs and ACs

The surface and pore structural properties of the resulting BCs and activated carbons are determined by N_2_ sorption-desorption at −196 °C using the Autosorb-1 MP (Kr-upgrade) gas sorption analyzer of Quantachrome. Before each measurement, all samples are outgassed at a high vacuum and a temperature of 250 °C for 24 h. As indicated from the results included in Table A4 and Table A5, BCs produced from SFD previously being washed with HNO_3_ 1% have a higher BET (in the range of 300–350 m^2^/g) than those produced from non-washed SFD (<50 m^2^/g). Washing the SFD with HNO_3_ 1% can increase the BET by at least 7 times (and up to 19 times) and the Total Pore Volume by at least 3 times (and up to 7 times), respectively. Pyrolysis temperature and residence time affect the material’s surface and pore characteristics; a higher pyrolysis temperature causes a higher BET and total pore volume, while longer pyrolysis residence time seems to not significantly affect porosity and surface characteristics.

**Figure 4:**
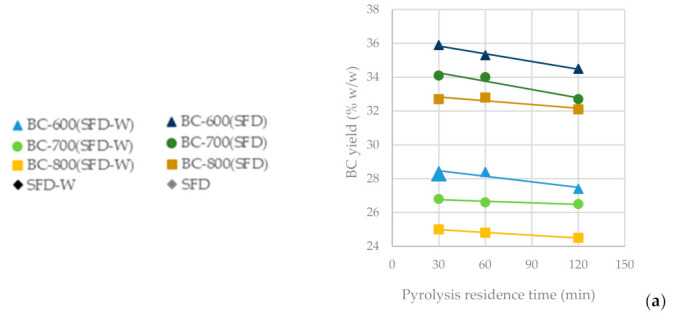

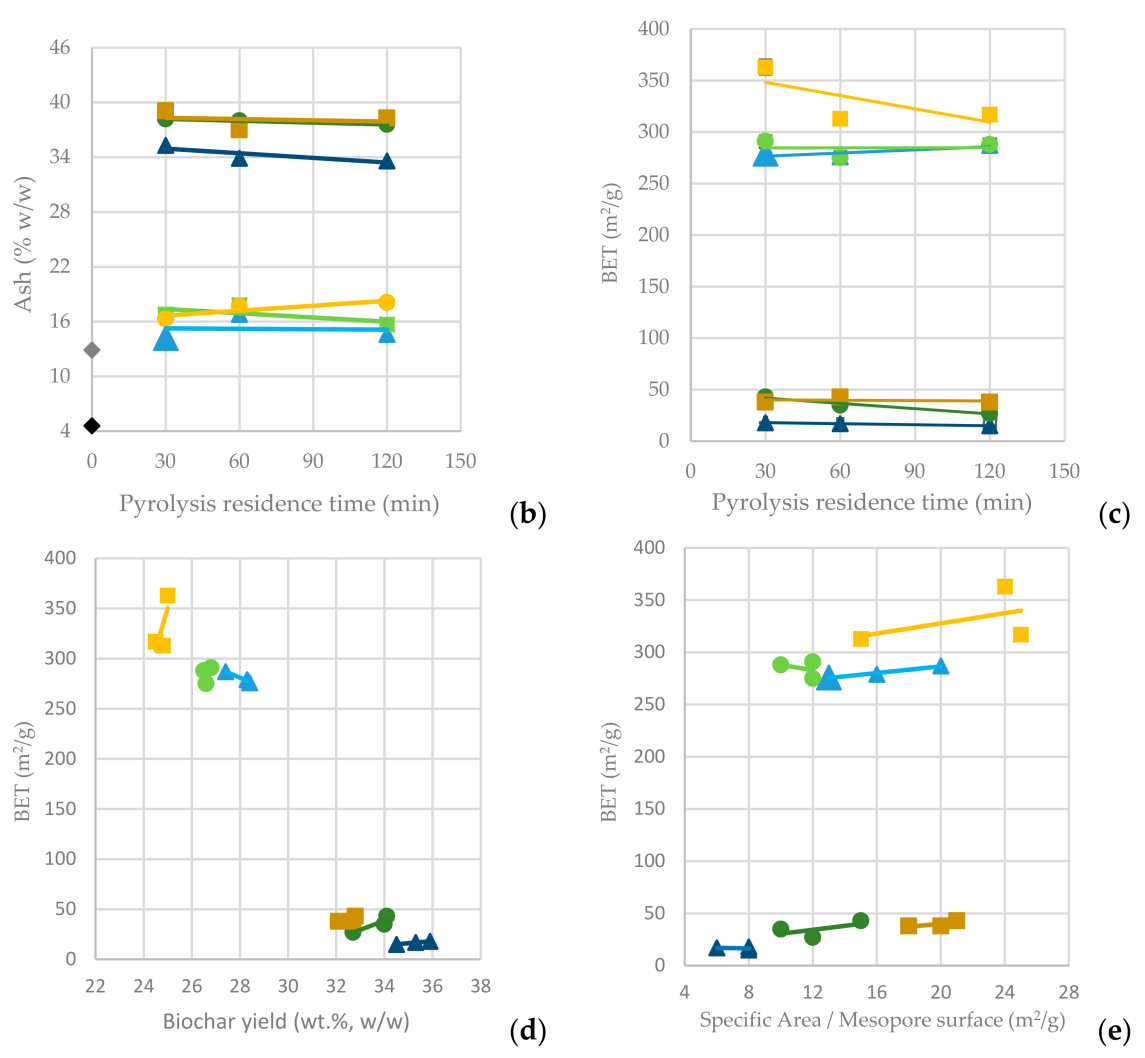
(**a**) BC yield vs. pyrolysis residence time (**b**) Ash content vs. pyrolysis residence time (**c**) BET surface area vs. pyrolysis residence time (**d**) BET surface area vs. BC yield (**e**) BET surface area vs. specific area/mesopore surface area from the pyrolysis of SFD and SFD-W at different pyrolysis conditions.

In Figure 4a, it is observed that as the pyrolysis temperature increases from 600 °C to 800 °C, the BC yield drops significantly (BC-SFD 32.10%; BC-SFD-W 24.50%), whereas the residence time seems not to significantly affect it. In Figure 4b, it can be observed that the ash content is affected inversely to the BC yield, meaning that the highest ash content (BC-SFD 39.10%; BC-SFD-W 18.10%) is achieved for the highest pyrolysis temperature (800 °C) and the lowest for the lowest pyrolysis temperature (600 °C), respectively, while the residence time does not significantly affect the ash content either. Aiming to obtain the most BC possible from the pyrolysis and considering (Figure 4c) that the BET is independent of whether the pyrolysis takes place at 600 or 700 °C, it becomes clear (Figure 4d,e) that a pyrolysis temperature of 600 °C serves both the purpose of achieving a high BC yield and a high BET, while the mesoporosity is also relatively high.

**Figure 5:**
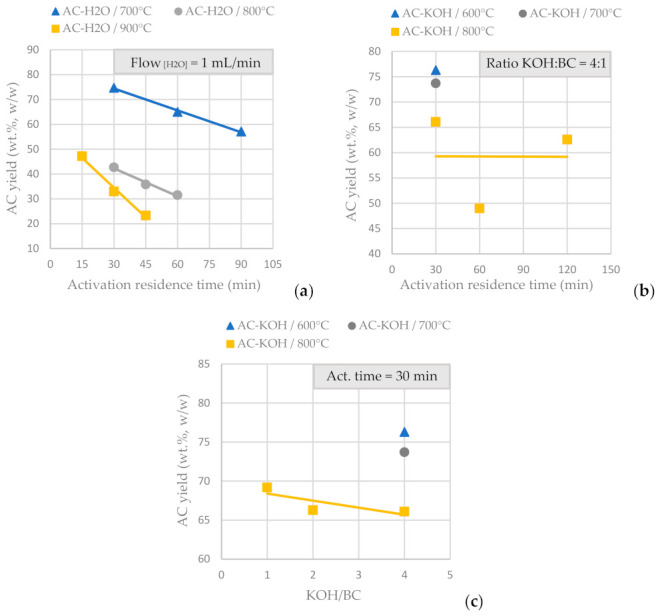
(**a**) AC yield vs. activation residence time for physical activation with steam, for a fixed H_2_O flow of 1mL/min at different activation temperatures, of the BC derived from the pyrolysis of SFD-W at 600 °C for 30 min. (**b**) AC yield vs. activation residence time for chemical activation with KOH, for a fixed KOH:BC ratio 4:1 at different activation temperatures, of the BC derived from the pyrolysis of SFD-W at 600 °C for 30 min (**c**) AC yield vs. KOH:BC ratio for chemical activation with KOH, for a fixed activation residence time of 30 min at different activation temperatures, of the BC derived from the pyrolysis of SFD-W at 600 °C for 30 min.

BCs obtained by SFD-W were transformed into activated carbon using physical activation with H_2_O (Table 4) and CO_2_ (50 mL/min at 850 °C and an activation duration of 150 min) and chemical activation with KOH (Table 5). Figure 5a presents the AC yield vs. the activation residence time for physical activation with fixed steam flow at 1mL/min and in Figure 5b presents the AC yield vs. the activation time for chemical activation with KOH at a fixed ratio of 4:1 over BC. It is concluded that for physical activation, prolonged residence time eliminates the AC yield (for a fixed steam flow), while for chemical activation, the AC yield is not affected significantly the longer the activation process lasts (for a fixed KOH/BC ratio). Figure 5c shows the AC yield vs. the KOH molar ratio for a fixed activation residence time; thus, it is concluded that the KOH molar ratio (from 1:1 to 4:1) doesn’t affect significantly the amount of AC produced from the functionalization process (for a fixed activation residence time). Temperature is the parameter that affects AC yield in both physical and chemical activation.

**Figure 6:**
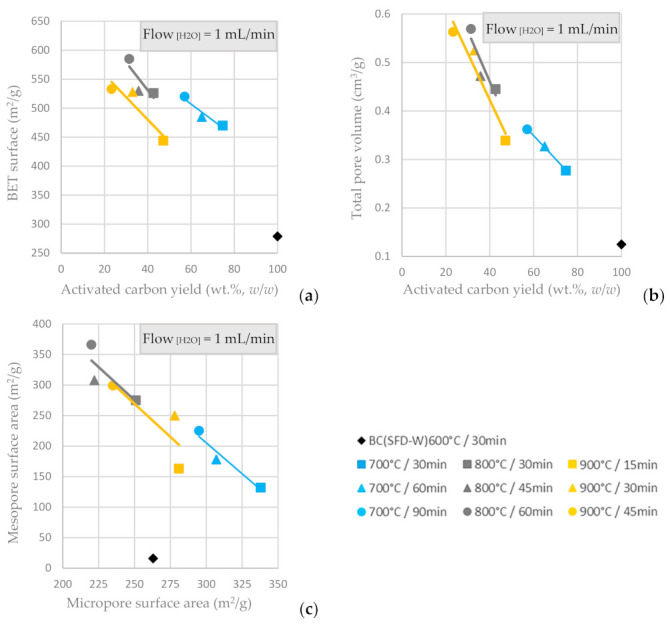
(**a**) BET surface area vs. AC yield wt.% *w*/*w* (**b**) Total pore volume vs. AC yield wt.% *w*/*w* of input BC. (**c**) Mesopore surface area vs. micropore surface area from the physical activation of BC-600 °C/30 min derived from the pyrolysis of SFD-W at different activation conditions.

In Figure 6a,b, the pore structural characteristics (BET and total pore volume) of the ACs produced via physical activation with steam are presented relative to the AC yield and the respective residence time. It is indicated that the BET surface area is enhanced for prolonged activation time, but an activation temperature higher than 800 °C does not offer any benefit to the BET and total pore volume values. The highest BET surface was achieved for an activation temperature of 800 °C and residence time of 60 min. Likewise, the total pore volume is optimized for the same conditions, concerning the physical activation with steam flow of 1 mL/min. Mesopore surface area is inversely proportional to the micropore surface area, as shown in Figure 6c and the highest mesopore surface area is achieved at activation temperature 800 °C and residence time of 60 min, while the highest micropore surface area is achieved at activation temperature 700 °C and residence time of 30 min (the lowest activation temperature and residence time tested). The steam-activated ACs had both micropores and mesopores, the amount of which could be tuned by adjusting the temperature and the duration of activation; a higher temperature and/or longer duration resulted in a decrease of the microporosity and an increase of mesoporosity.

**Figure 7:**
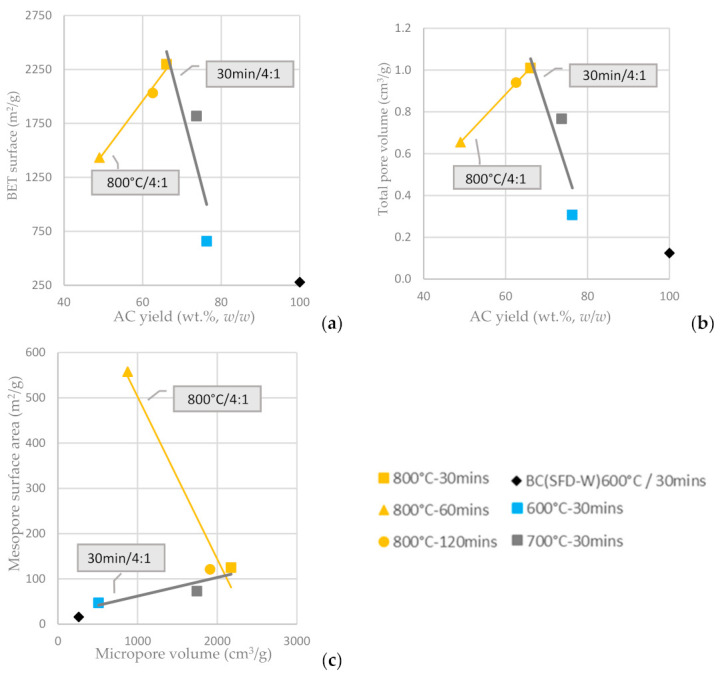
(**a**) BET surface area vs. AC yield wt.% *w*/*w* (**b**) Total pore volume vs. AC yield wt.% *w*/*w* of input BC (**c**) Mesopore surface area vs. micropore surface area from the physical activation of BC-600 °C/30 min derived from the pyrolysis of SFD-W at different activation conditions.

In Figure 7a, it is depicted that the BET surface area of the KOH-activated ACs (KOH:BC ratio of 4:1) is enhanced for longer residence time and is proportionally increased as long as AC yield is increased. On the other hand, for a fixed residence time and a fixed KOH:AC ratio, the BET surface area is increased as the activation temperature increases from 600 °C to 800 °C and the AC yield attenuates. The total pore volume of the KOH-activated ACs is affected in a similar way (Figure 7b). The mesopore surface area is, again, inversely proportional to the micropore surface area, as shown in Figure 7c and the highest mesopore surface area is achieved at activation temperature 800 °C and residence time of 60 min, while the highest micropore surface area is achieved at activation temperature 800 °C and residence time of 30 min (both at the highest activation temperature tested but different residence times). Furthermore, the chemically activated with KOH carbons had both micropores and mesopores, the amount of which could be also tuned by adjusting the temperature and the duration of activation; higher temperature resulted in an increase of both mesoporosity and microporosity, while the optimum residence time has been defined to be no more than 60 min.

**Figure 8:**
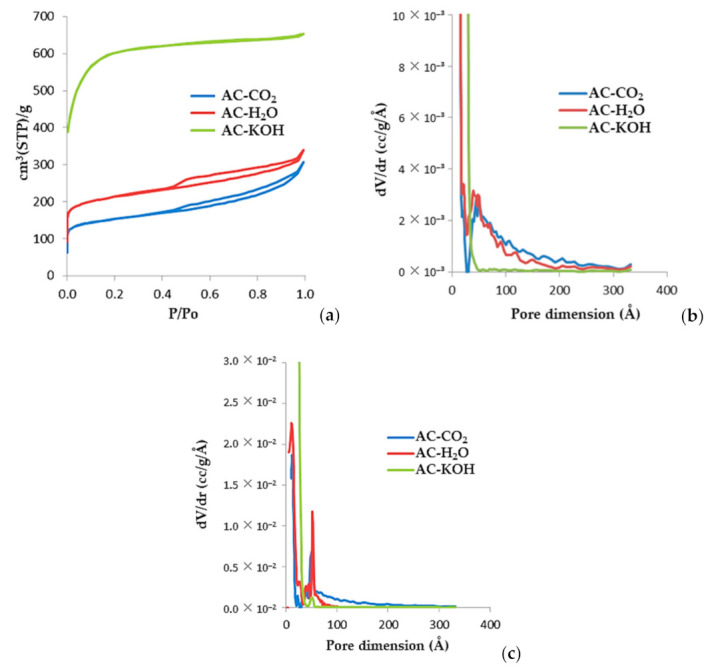
(**a**) N_2_ (77K) adsorption isotherms of porous carbons activated using various methods of physical and chemical activation. (**b**) QSDFT-derived pore size distributions obtained from the adsorption branch of the isotherms. (**c**) QSDFT-derived pore size distributions obtained from the desorption branch of the isotherms.

In Figure 8, we present the N_2_ (77K) adsorption/desorption isotherms and the respective pore size distribution of three selected samples, AC-H_2_O, AC-KOH, and AC-CO_2_). AC-H_2_O was produced with H_2_O activation for 60 min at 800 °C and a water vapor flow rate of 1 mL/min. Sample AC-KOH was produced via 30 min of chemical activation at a KOH molar ratio of 4:1 and temperature of 800 °C, while AC-CO_2_ was produced by physical activation at 850 °C for 150 min under a constant CO_2_ flow rate of 50 mL/min. The pore size distributions are derived with the QSDFT method for carbon and cylindrical pores, which is applied to both the adsorption and desorption branches of the isotherm. The micropore and external surfaces are presented in Table 7 and Table A4, Table A5, Table A6, Table A7 are derived from the analysis of the corresponding α_s_-plots using the N_2_ (77K) adsorption isotherm of a non-porous carbon as the reference. From the shape of the adsorption isotherms and the respective pore size distributions, it is concluded that physical activation of SFD-derived BC yields activated carbons with an extended mesopore structure. These carbons actually have a bimodal pore size distribution comprising micropores of the order of 12 Å and mesopores of the order of 50 Å. On the contrary, chemical activation with KOH leads to an almost purely microporous material with astonishing high surface area (2272 m^2^/g) and enhanced micropore volume of about 0.9 mL/g.

### 3.4. Surface Chemistry and Structural and Morphological Characteristics of ACs

**Figure 9:**
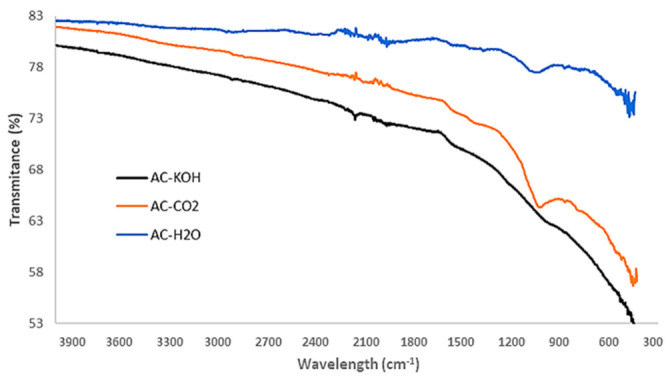
FTIR spectra of chemically and physically activated carbons.

Apart from pore structural characteristics, the surface chemistry of the activated carbons may have a significant effect on their gas adsorption capacity. In particular, the selectivity of CO_2_ over CH_4_ and of H_2_S over CO_2_ can be defined by the specific interactions of the gases with the functional groups sprawled on the pore surface of the AC samples. Hence, ATR-FTIR analysis is conducted with an Attenuated Total Reflectance (Brucker FTTR Spectrometer Alpha II, which features a monolithic diamond crystal) to detect and determine the several oxygenated functional groups that usually exist on the surface of ACs. The results presented in Figure 9 show that the physical activation methods, apart from being technically easy and sustainable, do not gravely affect the chemical composition of the formed carbons.

The peaks corresponding to C-O and C=O stretching vibrations are clearly distinguished in the AC-H_2_O and AC-CO_2_ samples whereas they are eliminated in the AC-KOH sample. This is an important result that will be discussed in the following section, in conjunction with the pore structural features of the AC samples and their capacity to selectively adsorb CO_2_ or H_2_S.

**Figure 10:**
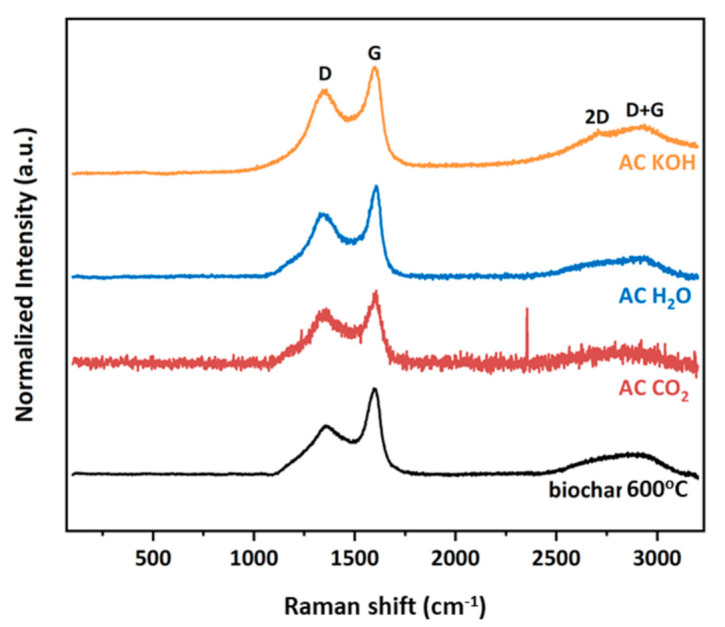
Raman spectra of all samples.

The Raman spectral analysis ranged from 100 to 3200 cm^−1^ in order to investigate the first- and second-orders of BC and the three derived activated carbons (ACs) samples. As depicted in Figure 10, the characteristic graphitic D and G peaks located at approximately 1375 and 1600 cm^−1^, respectively, are clearly observed for all studied materials. The D band is associated with a disordered graphitic structure, such as sp^3^ sites, and is denoted as a breathing mode with A_1g_ symmetry. On the other hand, the G band has E_2g_ symmetry corresponding to the in-plane stretching of sp^2^-bonded carbon atoms in a hexagonal graphitic ring. Furthermore, at this point, it could be mentioned that the intensity ratio of the D and G bands is a measure of the degree of disorder in a carbon material. For the precursor BC sample, the calculated I_D_/I_G_ value is 0.616, whereas for the three activated carbons, it is 0.830, 0.727 and 0.827 under CO_2_, H_2_O and KOH treatments, respectively. The similar trend of ratio increase for the activated carbons reveals the higher number of structural defects and disorder present compared to the precursor, but in general, for all studied samples, the I_D_/I_G_ ratio indicates similar obtained graphitic structure with formation of graphitized carbon and defects in lattice. The relatively broad D + G combination mode, due to the amorphous nature of samples in the range of 2800–2920 cm^−1^ is also presented for all samples. Finally, the 2D band, an overtone of the D band, is only observed in the AC KOH sample located at ~2700 cm^−1^. It is derived from a second-order Raman scattering process, and it is well-known that it is very sensitive to the stacking order of the graphene sheets. From its asymmetry and large Full Width at Half Maximum (FWHM), it is presumed that this sample has obtained a multi-layer graphitic structure.

**Figure 11:**
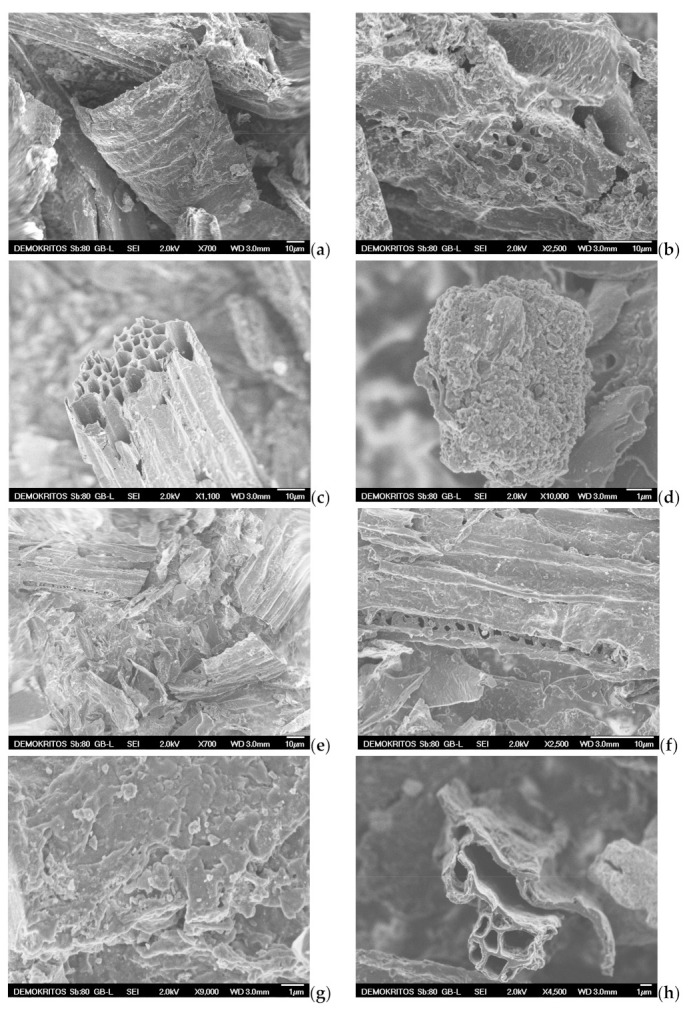

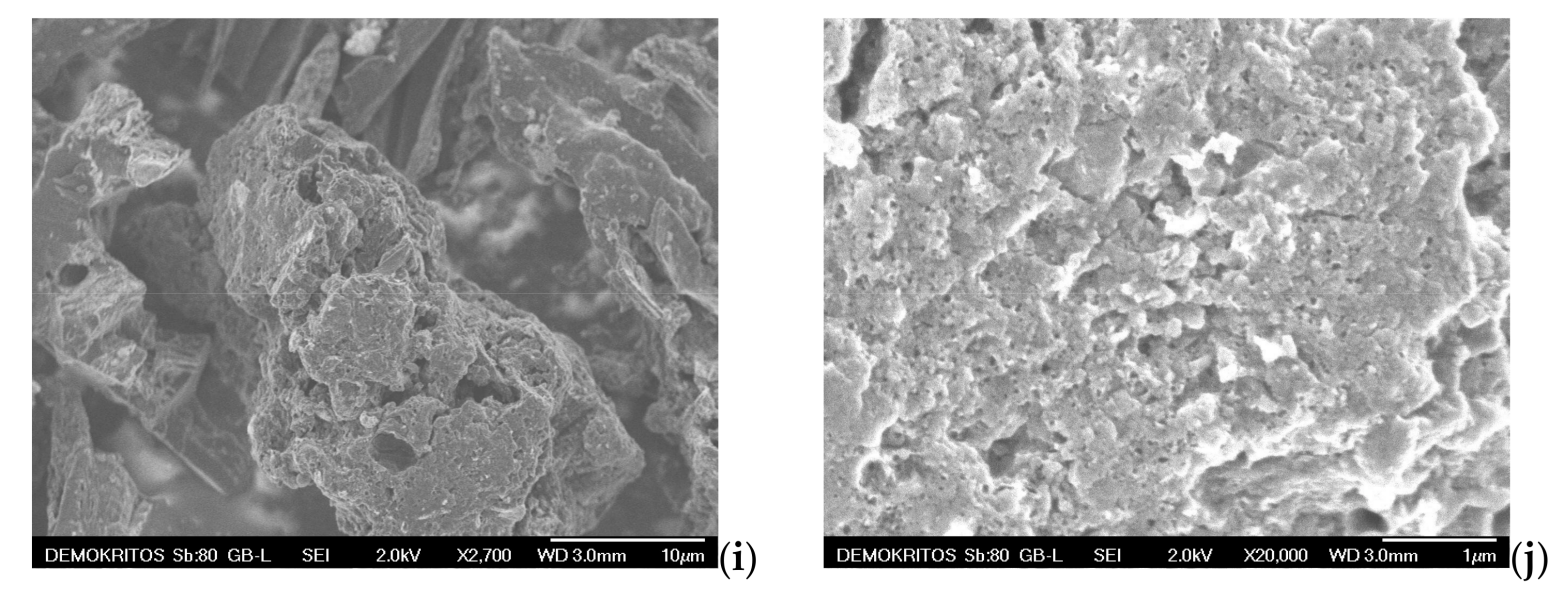
Scanning electron micrographs (SEM) of (**a**,**b**) BC-600 °C/30 min-SFD-W, (**c**,**d**) AC-H_2_O-800 °C/60 min/1 mL·min^−1^, (**e**–**g**) AC-KOH-800 °C/30 min/4:1, (**h**–**j**) AC-CO_2–_850 °C/150 min/50 mL·min^−1^.

SEM analysis is also applied to examine the surface morphology and possibly define the pore size and shape of both the precursor BC (Figure 11a,b) and the prepared ACs, namely the AC-H_2_O-800 °C/60 min/1 mL·min^−1^, the AC-KOH-800 °C/30 min/4:1 and the AC-CO_2_-850 °C/150 min/50 mL·min^−1^ as shown in Figure 11c–j. SEM images magnified by 700 to 1100 times visualize the presence of many honeycomb-shaped large pores (Figure 11c,h) for the ACs that are physically activated with steam and CO_2_, respectively. Regarding the chemically activated AC, a plate-form surface can be observed (Figure 11e), which is very similar to that of the BC precursor (Figure 11a). At higher magnifications (×2500–2700), well distributed macropores (pore widths of approximately 1.5–2 μm) can be distinguished on the sponge-like surface and plate-form surface of the ACs (Figure 11b,f,i). When the magnification goes beyond ×10,000, mesopores start to become distinguishable. Hence, in the CO_2_ activated AC, these mesopores are in the range of 10–50 nm (Figure 11j), while in the steam-activated AC (Figure 11d), the mesopores appearing on the sponge-like surface are a bit larger (40–80 nm), and some of them can be classified as small macropores. It is also clear that the population of mesopores on the surface of chemically activated AC is very low (Figure 11g). This indicates that activation with steam and CO_2_ results in more and well distributed mesopores of various sizes, while the chemical activation with KOH does not show such an intense population of mesopores or a homogeneous size distribution. Given the fact that the BET surface area and the micropore surface of AC-KOH are the highest measured, it is concluded that the microporosity of AC-KOH is readily enhanced (Table 7). However, micropores cannot be distinguished by SEM analysis (Figure 11j). Additionally, it can be seen that activation with CO_2_ has destroyed the honeycomb-shaped surface of the AC (Figure 11h), which must be caused by the prolonged residence time of activation (150 min).

### 3.5. Biogas Breakthrough Curves. Gas Separation Performance of the Developed ACs

**Figure 12:**
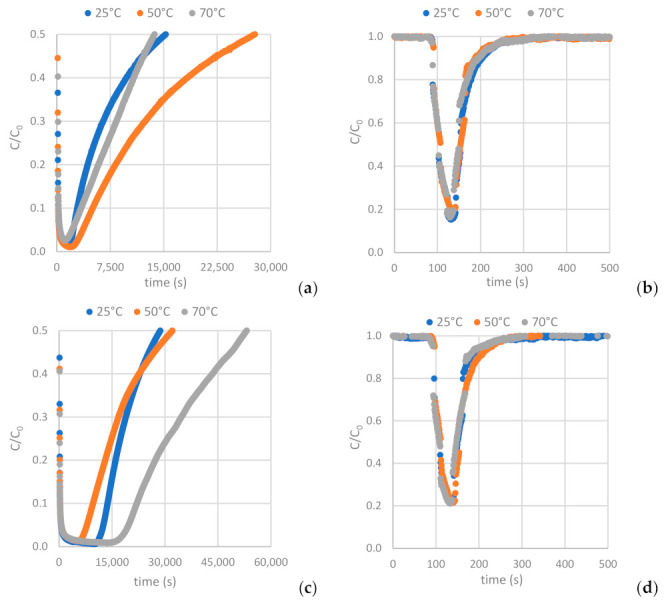

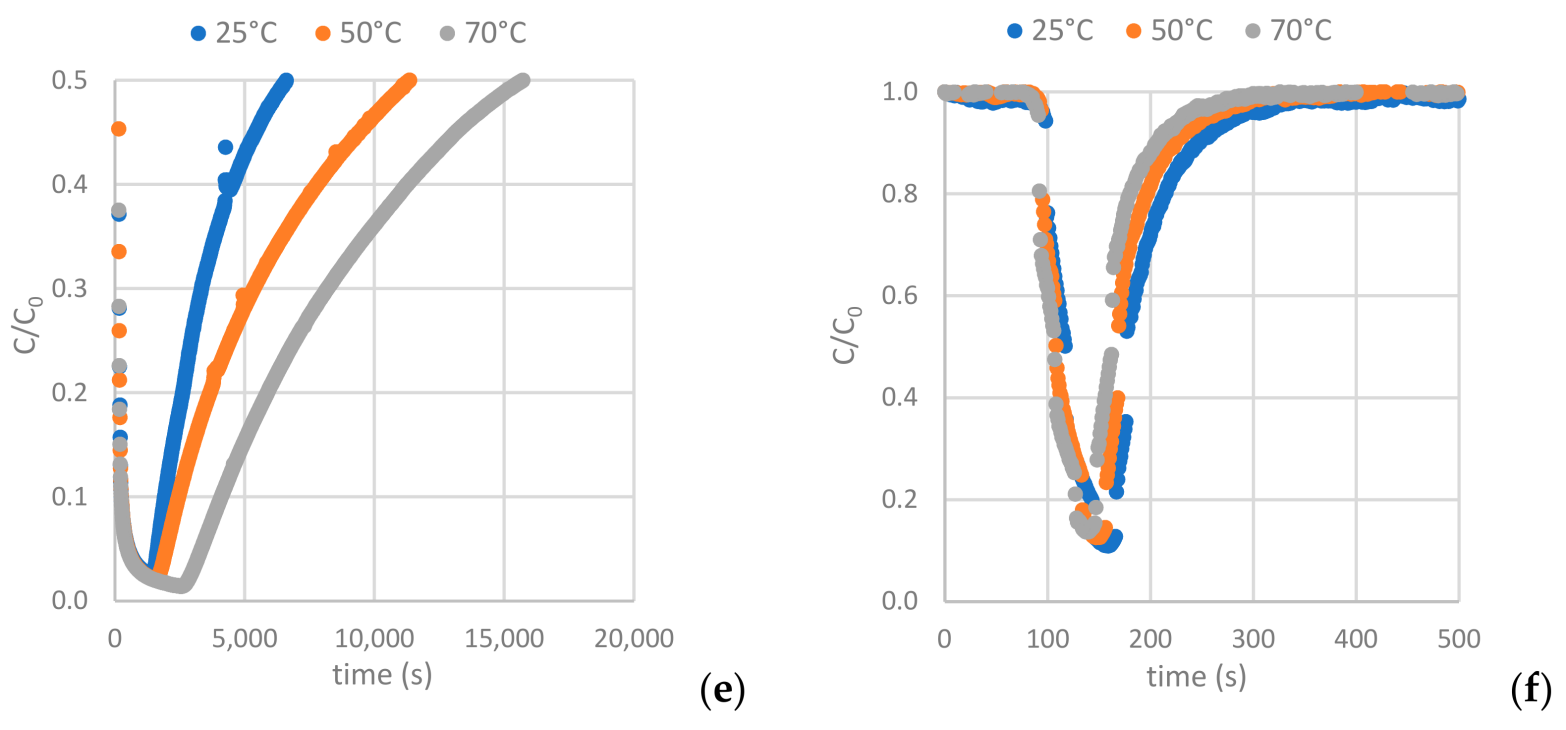
Breakthrough curves of: (**a**,**b**) H_2_S and CO_2_ on AC-H_2_O. (**c**,**d**) H_2_S and CO_2_ on AC-KOH. (**e**,**f**) H_2_S and CO_2_ on AC-CO_2_.

Figure 12 depicts the H_2_S and CO_2_ breakthrough curves obtained from samples AC-H_2_O, AC-CO_2_ and AC-KOH under the conditions described in Section 2.2.2 (Table 4 and Table 5). Having interpreted the breakthrough curves, the complete set of results is presented in Table 8 and Table 9 (see also Table A8 and Table A9).

**Figure 13:**
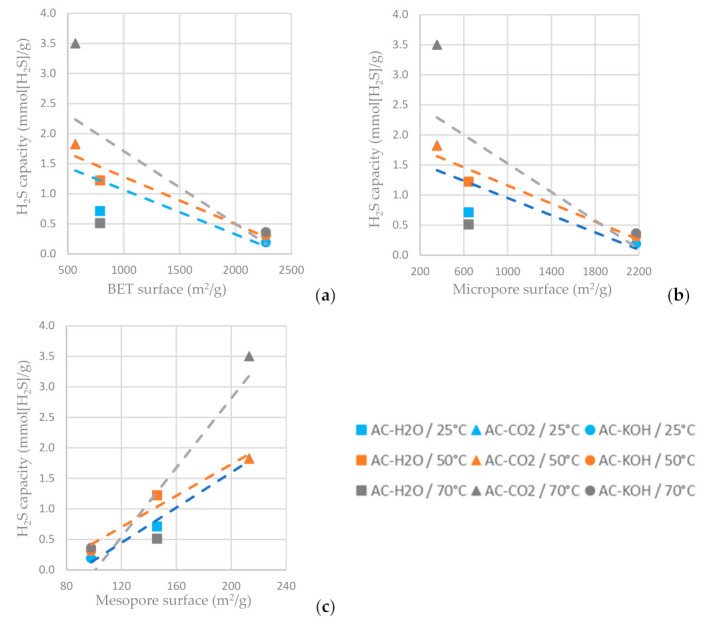
H_2_S capacity at time reference when C/C_0_ = 0.5 vs. (**a**) BET surface area, (**b**) micropore surface area and (**c**) mesopore surface area of the ACs functionalized with H_2_O, CO_2_ and CO_2_ at different temperatures.

As shown in Figure 13c, the higher mesopore area of the AC favors hydrogen sulfide adsorption, while the increased BET surface area and micropore area decrease the H2S sulfide capacity of the AC (Figure 13a,b). For the ACs tested, the one physically functionalized with CO_2_ has the highest hydrogen sulfide sorption capacity when the sorption temperature is settled at 70 °C. It is indicated in Figure 13 that a higher adsorption temperature favors H_2_S adsorption on the ACs.

**Figure 14:**
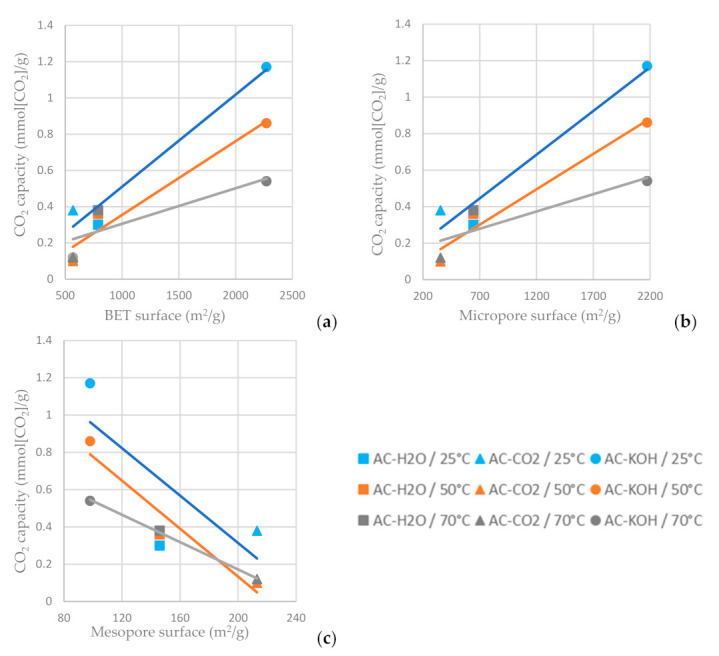
CO_2_ capacity at time reference when C/C_0_ = 0.95 vs. (**a**) BET surface area, (**b**) micropore surface area and (**c**) mesopore surface area of the ACs functionalized with H_2_O, CO_2_ and CO_2_ at different temperatures.

As shown in Figure 14a,b, the higher BET and micropore area of the AC favors carbon dioxide adsorption, while the increased mesopore area decreases the CO_2_ capacity of the AC (Figure 14c). For the ACs tested, the one chemically functionalized with KOH has the highest carbon dioxide sorption capacity when the sorption temperature is settled at 25 °C. It is indicated in Figure 14 that a lower adsorption temperature favors CO_2_ adsorption on the ACs.

The selectivity of hydrogen sulfide over carbon dioxide on the activated carbons tested has been calculated (Equation (1)) after obtaining the adsorbed amount results for both H_2_S and CO_2_ (Table 8 and Table 9). The results are displayed in Table 10, showing that the AC-CO2 is superior to the remaining ACs, resulting in a selectivity of at least 2 times more than the steam-activated ACs and more than 7 times higher than those chemically treated with KOH AC.

(1) SelectivityH2SCO2=qH2SPH2SqCO2PCO2

where $q_{H_{2}S}$:$q_{{CO}_{2}}$ adsorbed amount of H_2_S and CO_2_ and $P_{H_{2}S}$:$P_{{CO}_{2}}$ Partial pressure of H_2_S and CO_2_, respectively.

The results clearly show that the carbons derived from the physical activation of BC exhibit higher adsorption capacity for H_2_S and lower CO_2_ adsorptivity compared to the sample produced by the chemical activation method with KOH. A distinguishing feature of these samples is that, despite their moderate BET surface and micropore volume, they hold a quite extended mesopore structure with a PSD centered around 50 Å. In addition, contrary to what happens with the AC-KOH sample, the mesopores of the physically activated carbons preserve a high population of surface oxygenated functional groups. Hence, it becomes evident that H_2_S is strongly hindered from entering the micropore structure of ACs and is mostly adsorbed in the mesopores, also benefiting from its strong interaction with the functional groups. On the other hand, micropores are fully accessible for CO_2_, and this explains the much higher CO_2_ adsorptivity of AC-KOH as compared to AC-H_2_O and AC-CO_2_. Supporting these statements is that between the two physically activated samples, AC-CO_2_, despite its lower BET surface, is a more effective adsorbent for H_2_S because of its more extended mesopore structure (see Table 7) and possibly due to the higher population of oxygenated functional groups (Figure 4). Regarding the effect of temperature, in the case of H_2_S a maximum adsorptivity is observed systematically for all samples at 50 °C or a continuous increase up to 70 °C, something that does not happen with CO_2_, which in most of the experiments follows the normal trend of the adsorption exotherm. The maximum in H_2_S adsorptivity with temperature comes as a result of the counterbalance between adsorption and diffusion. This unveils that due to the strongly acidic character of H_2_S, its adsorptivity is controlled by diffusion, meaning that when the H_2_S molecules enter the pore, they reside for a long period on an adsorption site before hopping to the next unsaturated one. Diffusion is an activated process and is fortified as the temperature increases, whereas adsorption is attenuated. This counterbalance generates the maximum observed in our experiments.

## 4. Conclusions

This work concludes that in order to achieve the production of effective activated carbon adsorbents from the solid fibrous digestate (SFD) of biogas plants, washing with HNO_3_ to remove the inorganic content and expand the carbonaceous yield is a mandatory pre-treatment process. Moreover, BC intermediate can be produced by pyrolysis at moderate temperatures up to 600 °C with no effect on the quality of the subsequently derived ACs, which is of high importance for the sustainability of the proposed methodology. Notably, simple chemical and physical activation processes of the produced BCs conclude to very effective CO_2_ and H_2_S adsorbents respectively, paving the way for the achievement of a closed loop value chain where the waste effluent of a biogas plant is transformed to effective adsorbents that can be used in series to desulfurize and upgrade biogas.

## Figures and Tables

**Table 1:** Results obtained from cascade processes that combine adsorption and absorption columns for biogas treatment. Absorbers are filled with 1 L of solvent, while adsorber columns are filled with 500 g of adsorbent [1].

Gas	NaOH _(1.5 Molar)_+AC _(mass 500 g)_ +Steel Wool _(mass 500 g)_		Ca(OH)_2 (1 Molar)_ +Steel Wool _(mass 500 g)_	
	P: 2.5 cm of Hg	P: 5 cm of Hg	P: 2.5 cm of Hg	P: 7.5 cm of Hg
	Q: 10 LPM	Q: 2 LPM	Q: 10 LPM	Q: 2 LPM
CH_4_	+12%	+30%	+24%	+28%
CO_2_	−55%	−41%	−22%	−44%
H_2_S	−97%	−96%	−97%	−97%

**Table 2:** Details of the frequency of regeneration and related data for different reagents [1].

Reagent Chemical Formula	Cost (€)	Concentration of Aq. Solution or Adsorbent Mass	Volume of Biogas Purified before Saturation (m^3^)	Cost of Chemical for Purification (€/m^3^)
MEA, C_2_H_7_NO	6.21/L	10% *v*/*v*	165	Regeneration by heating
NaOH	1.24/kg	1.5 Molar	178	0.42
Granular AC	0.18/kg (limestone)	mass 100 g	117	0.12
Steel wool, Fe_2_O_3_	4.97/kg	mass 500 g	207	2.40
Ca(OH)_2_	0.2/kg	1 Molar	Regeneration up to 5 times	Regeneration by oxidization

**Table 3:** Moisture, ash content and elemental analysis of SFD and SFD-W.

	TS	Ash	C	H	N	O
	(wt.%, DM * feed)					
SFD	94.3	12.9	42.2	5.6	1.7	37.3
SFD-W	93	4.6	48.6	5.9	1.4	39.6

* on dry matter basis.

**Table 4:** Activated carbon yield (wt.%, dry matter feed), with physical activation (H_2_O) at various activation temperatures, steam flow and activation time.

Activation Temperature	Steam Flow	Activation Time	AC Yield (wt.%, DM * Feed)
(°C)	(mL/min)	(min)	AC-H_2_O
700	1	30	74.7
700	1	60	65.0
700	1	90	57.1
800	1	30	42.7
800	1	45	35.8
800	1	60	31.5
900	1	15	47.2
900	1	30	33.0
900	1	45	23.3

* on dry matter basis.

**Table 5:** Activated carbon yield (wt.%, dry matter feed), with chemical activation (KOH) at various activation temperatures, reagent KOH to BC ratio and activation time.

Activation Temperature	Ratio KOH/BC	Activation Time	AC Yield (wt.%, DM * Feed)
(°C)		(min)	AC-KOH
600	4	30	76.3
700	4	30	73.7
800	1	30	69.2
800	2	30	66.3
800	4	30	66.1
800	4	60	49.0
800	4	120	62.6

* on dry matter basis.

**Table 6:** Experimental conditions of the biogas breakthrough tests.

Activated Carbon	Reactor Temperature (°C)	Flow Characteristics					
		Q_biogas_ (mL/min)	ΔΡ_biogas_ (millibar)	Density (g/cm^3^)	V_average_ (cm/min)	η_biogas_ (Poise)	H (mm)
AC-H_2_O	25	39.75	0.0401	0.0423	219.78	9.97 × 10^−6^	1034.54
	50	40.03	0.0447	0.0445	221.33	1.10 × 10^−5^	975.78
	70	39.10	0.0472	0.0449	216.18	1.19 × 10^−5^	934.02
AC-KOH	25	39.77	0.0401	0.0423	219.89	9.97 × 10^−6^	1034.54
	50	39.85	0.0445	0.0443	220.33	1.10 × 10^−5^	975.78
	70	39.73	0.0480	0.0457	219.67	1.19 × 10^−5^	934.02
AC-CO_2_	25	40.12	0.0404	0.0426	221.82	9.97 × 10^−6^	1034.54
	50	40.05	0.0447	0.0445	221.44	1.10 × 10^−5^	975.78
	70	39.02	0.0471	0.0448	215.74	1.19 × 10^−5^	934.02

**Table 7:** Surface characteristics and porosity of activated carbons AC-H_2_O, AC-KOH and AC-CO_2_ produced by activation of BC, which has been derived from the pretreated SFD.

Sample	Activation Agent	BET	Micropore Surface	External Surface	Micropore Volume	Total Pore Volume
		(m^2^/g)	(m^2^/g)	(m^2^/g)	(cm^3^/g)	(cm^3^/g)
BC-SFD-W	-	279	263	16	0.103	0.125
AC-H_2_O	H_2_O	790	644	146	0.31	0.53
AC-CO_2_	CO_2_	568	355	213	0.224	0.48
AC-KOH	KOH	2272	2174	98	0.89	1.011

**Table 8:** H_2_S adsorption capacity, molar mass of H_2_S adsorbed per AC mass, on the activated carbon materials at various temperatures.

Activated Carbon	Adsorption Temperature (°C)	H_2_S Capacity (mmol[H_2_S]/g)		
		Breakthrough Time (C/C_0_ = 0.05) ^1^	Reference Time C/C_0_ = 0.5 ^1^	Exhaustion Time (C/C_0_ = 0.95) ^2^
AC-H_2_O	25	0.10	0.71	2.13
	50	0.09	1.22	3.26
	70	0.04	0.51	1.54
AC-KOH	25	0.05	0.19	0.88
	50	0.05	0.31	1.42
	70	0.07	0.36	0.79
AC-CO_2_	25	0.75	1.83	4.70
	50	0.36	1.82	4.63
	70	0.86	3.50	7.51

^1^ Experimental. ^2^ Calculated.

**Table 9:** CO_2_ adsorption capacity, molar mass of CO_2_ adsorbed per AC mass, on the activated carbon materials at various temperatures.

Activated Carbon	Adsorption Temperature (°C)	CO_2_ Capacity (mmol[CO_2_]/g)			
		Breakthrough Time (C/C_0_ = 0.05) ^1^	C/C_0_ = 0.5 ^1^	Exhaustion Time (C/C_0_ = 0.95) ^1^	Reference Time C/C_0_ = 0.5 ^2^
AC-H_2_O	25	0.08	0.19	0.30	0.30
	50	0.00	0.15	0.36	0.36
	70	0.11	0.20	0.38	0.38
AC-KOH	25	0.21	0.62	1.17	1.17
	50	0.13	0.49	0.86	0.86
	70	0.04	0.37	0.54	0.54
AC-CO_2_	25	0.07	0.17	0.38	0.38
	50	0.06	0.10	0.10	0.10
	70	0.05	0.11	0.12	0.12

^1^ CO_2_ breakthrough time, time when C/C_0_ = 0.5 and exhaustion time. ^2^ Reference time; when C/C_0_ = 0.5 at the adsorption curve of H_2_S.

**Table 10:** Selectivity of H_2_S over CO_2_ on activated carbon materials at various temperatures.

Activated Carbon	Reactor Temperature (°C)	Selectivity of H_2_S		
		Breakthrough Time (C/C_0_ = 0.05) ^1^	Reference Time C/C_0_ = 0.5 ^1^	Exhaustion Time (C/C_0_ = 0.95) ^2^
AC-H_2_O	25	254	1903	5680
	50	204	2719	7227
	70	86	1081	3248
AC-KOH	25	34	132	606
	50	45	288	1331
	70	106	525	1166
AC-CO_2_	25	1573	3810	9795
	50	2902	14,606	37,075
	70	5607	22,859	49,010

^1^ Experimental. ^2^ Calculated.

## Data Availability

The data presented in this study are available upon request from the corresponding author.

## Appendix A

**Table A1:** BC yield (wt.%, dry matter feed) at various pyrolysis temperatures and times.

Pyrolysis Temperature	Pyrolysis Time	BC Yield (wt.%, DM * Feed)	
(°C)	(min)	BC-SFD	BC-SFD-Washed
600	30	35.90	28.3
600	60	35.30	28.4
600	120	34.50	27.4
700	30	34.10	26.8
700	60	34.00	26.6
700	120	32.70	26.5
800	30	32.70	25.0
800	60	32.80	24.8
800	120	32.10	24.5

* on dry matter basis.

**Table A2:** Ash content and elemental analysis of BC materials derived from the untreated SFD.

Pyrolysis Temperature	Pyrolysis Time	Ash	C	H	N	O	H/C	O/C
(°C)	(min)	(wt.%, DM * Feed)						
600	30	35.30	58.80	1.60	0.50	3.70	0.33	0.05
600	60	33.90	58.60	1.60	0.80	5.10	0.32	0.07
600	120	33.60	61.40	1.30	0.90	2.80	0.25	0.03
700	30	38.20	58.70	1.10	0.50	1.40	0.23	0.02
700	60	38.00	58.90	1.30	0.70	1.20	0.27	0.02
700	120	37.60	60.90	0.90	0.70	0.00	0.18	0.00
800	30	39.10	61.10	0.70	0.30	0.00	0.14	0.00
800	60	37.00	61.20	0.60	0.50	0.70	0.12	0.01
800	120	38.30	60.30	0.40	0.90	0.10	0.07	0.00

* on dry matter basis.

**Table A3:** Ash content and elemental analysis of BC materials derived from the pre-treated SFD.

Pyrolysis Temperature	Pyrolysis Time	Ash	C	H	N	O	H/C	O/C
(°C)	(min)	(wt.%, DM * Feed)						
600	30	14.20	77.70	2.20	0.60	5.10	0.35	0.05
600	60	16.80	76.20	1.90	1.30	3.80	0.31	0.04
600	120	14.60	80.60	1.70	0.00	3.10	0.25	0.03
700	30	16.80	77.20	1.80	1.20	3.00	0.28	0.03
700	60	17.80	77.70	1.50	1.10	1.80	0.24	0.02
700	120	15.70	78.30	1.60	1.00	3.50	0.24	0.03
800	30	16.30	86.80	0.70	1.40	0.00	0.09	0.00
800	60	17.70	78.80	0.90	0.00	2.50	0.14	0.02
800	120	18.10	82.30	0.60	0.90	0.00	0.09	0.00

* on dry matter basis.

**Table A4:** Surface characteristics and porosity of BC materials derived from the untreated SFD.

Pyrolysis Temperature	Pyrolysis Time	BET	Micropore Surface	External Surface	Micropore Volume	Total Pore Volume
(°C)	(min)	(m^2^/g)	(m^2^/g)	(m^2^/g)	(cm^3^/g)	(cm^3^/g)
600	30	18	10	8	0.004	0.021
600	60	17	11	6	0.004	0.019
600	120	15	7	8	0.003	0.020
700	30	43	28	15	0.011	0.040
700	60	35	25	10	0.010	0.035
700	120	27	16	12	0.006	0.034
800	30	38	18	20	0.007	0.043
800	60	43	22	21	0.009	0.056
800	120	38	20	18	0.008	0.055

**Table A5:** Surface characteristics and porosity of BC materials derived from the pretreated SFD.

Pyrolysis Temperature	Pyrolysis Time	BET	Micropore Surface	External Surface	Micropore Volume	Total Pore Volume
(°C)	(min)	(m^2^/g)	(m^2^/g)	(m^2^/g)	(cm^3^/g)	(cm^3^/g)
600	30	279	263	16	0.103	0.125
600	60	276	265	13	0.103	0.129
600	120	287	268	20	0.104	0.135
700	30	291	279	12	0.108	0.127
700	60	275	263	12	0.102	0.122
700	120	288	278	10	0.107	0.125
800	30	363	339	24	0.132	0.165
800	60	313	298	15	0.115	0.138
800	120	317	292	25	0.113	0.151

**Table A6:** Surface characteristics and porosity of activated carbons AC-H_2_O produced by activation of BC derived from the pretreated SFD.

Activation Temperature	Steam Flow Rate	Activation Time	BET	Micropore Surface	External Surface	Micropore Volume	Total Pore Volume
(°C)	(mL/min)	(min)	(m^2^/g)	(m^2^/g)	(m^2^/g)	(cm^3^/g)	(cm^3^/g)
700	1	30	470	338	132	0.139	0.277
700	1	60	485	307	178	0.129	0.327
700	1	90	520	295	225	0.125	0.362
800	1	30	526	251	275	0.107	0.445
800	1	45	530	222	308	0.095	0.472
800	1	60	585	220	366	0.095	0.569
900	1	15	444	281	163	0.116	0.339
900	1	30	528	278	250	0.117	0.525
900	1	45	533	235	299	0.100	0.563

**Table A7:** Surface characteristics and porosity of activated carbons AC-KOH produced by activation of BC derived from the pretreated SFD.

Activation Temperature	KOH Molar Ratio	Activation Time	BET	Micropore Surface	External Surface	Micropore Volume	Total Pore Volume
(°C)		(min)	(m^2^/g)	(m^2^/g)	(m^2^/g)	(cm^3^/g)	(cm^3^/g)
600	4	30	658	511	47	0.246	0.307
700	4	30	1818	1746	73	0.688	0.767
800	1	30	1196	1143	52	0.446	0.502
800	2	30	367	335	32	0.134	0.178
800	4	30	2299	2174	125	0.887	1.010
800	4	60	1434	876	558	0.341	0.655
800	4	120	2032	1911	121	0.779	0.940

**Table A8:** H_2_S adsorption capacity, mass of H_2_S adsorbed per AC mass, on the activated carbon materials at various temperatures.

Activated Carbon	Adsorption Temperature (°C)	H_2_S Capacity (g [H_2_S]/g)		
		Breakthrough Time (C/C_0_ = 0.05) ^1^	Reference Time C/C_0_ = 0.5 ^1^	Exhaustion Time (C/C_0_ = 0.95) ^2^
AC-H_2_O	25	2.79	20.93	62.47
	50	2.70	35.94	95.53
	70	1.20	15.02	45.13
AC-KOH	25	1.46	5.66	25.95
	50	1.40	9.03	41.75
	70	2.12	10.47	23.23
AC-CO_2_	25	22.14	53.62	137.83
	50	10.62	53.44	135.65
	70	25.19	102.69	220.17

^1^ Experimental. ^2^ Calculated.

**Table A9:** CO_2_ adsorption capacity, mass of CO_2_ adsorbed per AC mass, on the activated carbon materials at various temperatures.

Activated Carbon	Adsorption Temperature (°C)	CO_2_ Capacity (g [CO_2_]/g)			
		Breakthrough Time (C/C_0_ = 0.05) ^1^	C/C_0_ = 0.5 ^1^	Exhaustion Time (C/C_0_ = 0.95) ^1^	Reference Time C/C_0_ = 0.5 ^2^
AC-H_2_O	25	1.82	4.32	6.82	6.82
	50	0.00	3.41	8.18	8.18
	70	2.50	4.54	8.63	8.63
AC-KOH	25	4.77	14.09	26.58	26.58
	50	2.95	11.13	19.54	19.54
	70	0.91	8.41	12.27	12.27
AC-CO_2_	25	1.59	3.86	8.63	8.63
	50	1.36	2.27	2.27	2.27
	70	1.14	2.50	2.73	2.73

^1^ CO_2_ breakthrough time, time when C/C_0_ = 0.5 and exhaustion time. ^2^ Reference time; when C/C_0_ = 0.5 at the adsorption curve of H_2_S.
